# Creation of Recombinant Biocontrol Agents by Genetic Programming of Yeast

**DOI:** 10.32607/actanaturae.11878

**Published:** 2023

**Authors:** S. O. Pipiya, N. Z. Mirzoeva, M. N. Baranova, I. E. Eliseev, Yu. A. Mokrushina, O. V. Shamova, A. G. Gabibov, I. V. Smirnov, S. S. Terekhov

**Affiliations:** Institute of Bioorganic Chemistry, Russian Academy of Sciences, academicians M.M. Shemyakin and Yu.A. Ovchinnikov, Russian Academy of Sciences, Moscow, 117997 Russian Federation; Lomonosov Moscow State University M.V. Lomonosov, Moscow, 119234 Russian Federation; Institute of Experimental Medicine, St. Petersburg, 197022 Russian Federation; Federal State Budgetary Institution “National Medical Research Center of Endocrinology” of the Ministry of Health of the Russian Federation, Moscow, 115478 Russian Federation

**Keywords:** Antimicrobial peptides (AMPs), yeast Pichia pastoris, heterologous expression, protegrin-1 (PG-1), microfluidic compartmentalization, emulsion microcultivation

## Abstract

Bacterial infections caused by antibiotic-resistant pathogens pose an extremely serious and elusive problem in healthcare. The discovery and targeted creation of new antibiotics are today among the most important public health issues. Antibiotics based on antimicrobial peptides (AMPs) are of particular interest due to their genetically encoded nature. A distinct advantage of most AMPs is their direct mechanism of action that is mediated by their membranolytic properties. The low rate of emergence of antibiotic resistance associated with the killing mechanism of action of AMPs attracts heightened attention to this field. Recombinant technologies enable the creation of genetically programmable AMP producers for large-scale generation of recombinant AMPs (rAMPs) or the creation of rAMP-producing biocontrol agents. The methylotrophic yeast Pichia pastoris was genetically modified for the secreted production of rAMP. Constitutive expression of the sequence encoding the mature AMP protegrin-1 provided the yeast strain that effectively inhibits the growth of target gram-positive and gram-negative bacteria. An antimicrobial effect was also observed in the microculture when a yeast rAMP producer and a reporter bacterium were co-encapsulated in droplets of microfluidic double emulsion. The heterologous production of rAMPs opens up new avenues for creating effective biocontrol agents and screening antimicrobial activity using ultrahigh-throughput technologies.

## INTRODUCTION

Antibiotic resistance (AR) poses a major challenge to the global healthcare system. According to some estimates, infections caused by antimicrobial-resistant bacterial strains were responsible for the death of 4.95 million people in 2019 [1]. The number of strains acquiring resistance to antibiotics, including last-resort ones, is also increasing. Yet, the number of novel antibacterial agents approved for clinical use continues to decrease with every year, in opposition to the AR spread rate [2], which makes it necessary to search for alternative approaches to infectious disease control.

The global community has identified the top-priority pathogens that necessitate prompt action to develop novel approaches aimed at combating them [3]. These pathogens are known under the acronym "ESKAPE" and include such bacteria as Enterococcus faecium, Staphylococcus aureus, Klebsiella pneumoniae, Acinetobacter baumannii, Pseudomonas aeruginosa, and species that belong to the genus Enterobacter. Antimicrobial peptides (AMPs) are particularly effective against bacterial infections caused by antibioticresistant members of this group of bacteria [4]. AMPs are produced by a broad range of organisms and exhibit antibacterial, antifungal, and immunomodulatory activities [5]. The mechanisms of action and the molecular targets of AMPs differ from the targets of low-molecular-weight antibiotics. AMPs are often membrane-targeting; they form pores in the lipid bilayer or affect cell wall biosynthesis, thus disrupting the integrity of bacterial cells and causing pathogen death [6]. Due to this mechanism of action, bacteria develop lower resistance to AMPs [7, 8].

A limited number of AMPs is currently available for therapeutic use; however, the number of AMPs undergoing preclinical and clinical trials is increasing, thus proving that this field of research is very promising [9, 10]. The cost of AMP production by solid-phase synthesis can be as high as US$ 50–400 per gram of the product, which is economically feasible mainly for short peptides [11]. Furthermore, chemical synthesis technologies do not allow one to perform large-scale screening of antimicrobial activity employing the principles of combinatorial chemistry and biology [12]. An alternative approach is to use heterologous systems for recombinant production of AMPs. The heterologous production systems based on the methylotrophic yeast Pichia pastoris allow one to easily scale up manufacturing of recombinant biologics, thus minimizing their production costs [13, 14].

**Fig. 1:**
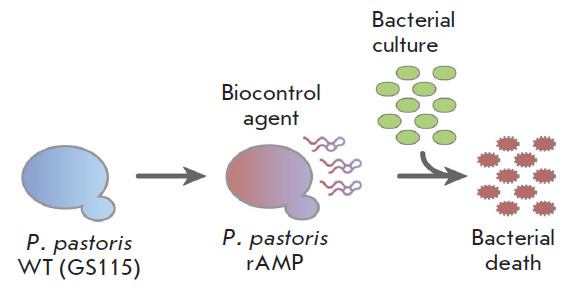
Schematic representation of the genetic programming of *P. pastoris *yeast strains and the creation of a recombinant biocontrol agent: wild-type yeast (*P. pastoris *WT GS115) is transfected with a genetic construct for the secretory production of AMP, and cocultivation of the resulting biocontrol agent (*P. pastoris *rAMP) with a target bacterium leads to bacterial elimination

Biocontrol agents are living organisms, either natural or modified, that can inhibit the spread of pathogens and harmful organisms [15]. This term is most often used in the context of biopesticide design. Since yeast cells are not the targets of most AMPs, they can be used to create biocontrol agents that secrete active AMPs into the extracellular environment in order to inhibit the growth of pathogenic bacteria (Fig. 1) [16] or phytopathogenic fungi [17]. The application of this approach for controlling pathogens, including those from the ESKAPE group, can be promising in limiting the spread of antibiotic resistance.

This study focuses on the genetic programming of the methylotrophic yeast P. pastoris in order to generate recombinant biocontrol agents, with antimicrobial peptide acting as the active component. A genetic construct ensuring constitutive production of mature AMP secreted into the culture medium was generated. The yeast P. pastoris transfected with this construct exhibited antimicrobial activity against both gram-negative and gram-positive target bacteria. A significant antimicrobial effect was also observed in the emulsion microculture mimicking natural bacterial microcompartments. Co-encapsulation of target bacteria and AMP-secreting yeast cells in droplets of microfluidic double emulsion effectively inhibited bacterial growth through the heterologous production of rAMP, protegrin-1 (rPG-1). The elaborated approach to designing recombinant biocontrol agents is rather promising for further development of alternative strategies to combat antibiotic resistance.

## EXPERIMENTAL

**Bacterial and yeast strains**

The P. pastoris GS115 strain (Invitrogen, U.S.) was used as a heterologous producer of AMP. Antimicrobial activity was checked for the bacterial strains Escherichia coli ΔlptD (kindly provided by I.A. Osterman) and Bacillus megaterium B-512 (kindly provided by S.A. Dubiley). For generating the E. coli ΔlptD sfGFP reporter strain, E. coli ΔlptD cells were transfected with a plasmid that constitutively expressed the green fluorescent protein sfGFP [18].

**Plasmid construction and transfection into yeast cells**

The codons of the sequence coding for recombinant protegrin-1 (rPG-1) were optimized using the GeneArt GeneOptimizer software (Thermo Fisher Scientific Inc., U.S.). The optimized rPG-1 gene sequence was cloned into the pGAPZalpha A expression vector (Thermo Fisher Scientific Inc.) by homologous recombination. The resulting pGAP-PG-1 plasmid was linearized at the AvrII restriction site and transfected into yeast cells by electroporation [19]. The transfected clones were chosen on selective YPDS agar medium (2% peptone, 1% yeast extract, 2% glucose, 1 M sorbitol, 2% agar) supplemented with the zeocin antibiotic until the final concentration of 100 μg/mL was attained.

**Analysis of the growth inhibition zones of target bacteria**

In order to measure the diameter of the growth inhibition zones, P. pastoris clones were cultured in plates containing YPD agar (1% yeast extract, 2% peptone, 2% glucose, 100 mM potassium phosphate pH 6.0, and 1.8% agar) during 2 days at 30°C. Soft agar (8 g/L tryptone, 2.5 g/L NaCl, 5 g/L yeast extract, and 0.5% agar) was melted, cooled down to 42°C, and E. coli ΔlptD or B. megaterium B-512 was inoculated until a final concentration of ~106 CFU/mL. The P. pastoris colonies were then covered with inoculated soft agar and incubated at 37°C overnight. The presence of antimicrobial activity was analyzed based on the diameter of the growth inhibition zones of the reporter bacterium.

**Estimation of the concentration of recombinant protegrin-1 in the culture medium**

The rPG-1 producer yeast strain was cultured in the YPD medium (1% yeast extract, 2% peptone, 2% glucose, and 100 mM potassium phosphate pH 6.0) in shake flasks at 37°C and 250 rpm during 3 days. The culture medium was used to analyze the antimicrobial activity against the target bacterium E. coli ΔlptD using the two-fold serial dilution method. A synthetic analog of protegrin-1 produced by solid-phase synthesis was used as a reference standard for determining the peptide concentration.

**Encapsulation of yeast strains and the target bacterium into droplets of microfluidic double emulsion and flow cytometry**

The reporter strain E. coli ΔlptD sfGFP producing sfGFP under control of the pJ23119 promoter was cultured in the YPD medium (1% yeast extract, 2% peptone, 2% glucose, and 100 mM potassium phosphate pH 6.0) in shake flasks at 37°C and 250 rpm until they reached the logarithmic growth phase. P. pastoris GS115 and rPG-1 were cultured in the YPD medium in shake flasks at 30°C and 180 rpm during 16 h. Next, the cell cultures were filtered using 40 μm cell strainers (Greiner Bio-One, Germany) and diluted to the optical densities of OD_600_ = 0.45 (occupancy (λ) ~ 5 cells per droplet) for E. coli ΔlptD and OD_600_ = 1.5 (occupancy (λ) ~ 1 cell per droplet) for the yeast strains. The cells were then encapsulated into droplets of microfluidic double emulsion (MDE) using 20 µm microfluidic chips produced by soft lithography according to the procedure described previously [20]. The filled MDE droplets were cultured at 30°C in an incubator saturated with water vapor. After incubation for 24 h, the fluorescence signal from the MDE droplets was analyzed using a Novocyte Flow Cytometer system (ACEA Biosciences Inc., USA). The droplets were visualized using an Eclipse Ti inverted fluorescent microscope (Nikon, Japan) with the standard FITC filter. The experiment involving cocultivation of yeasts and bacteria in a 96-well plate was conducted in the YPD medium with the initial optical densities OD_600_ = 0.25 for yeasts and OD_600_ = 0.005 for E. coli ΔlptD sfGFP. The plate was incubated at 30°C under constant stirring. The growth of the target bacterium was assessed by counting colonies after inoculation of serial ten-fold dilutions of the coculture onto an agar medium. The measurements were performed in three replicates.

## RESULTS

**Antimicrobial peptides as effective antibacterial agents**

Antimicrobial peptides can be simultaneously characterized by a high antimicrobial efficacy and a broad spectrum of antimicrobial activity. Table 1 summarizes the results of our analysis of the published data on the antimicrobial activity of a number of highly active AMPs.

Highly effective AMPs can belong to different structural classes. Protegrin-1 and arenicin-1 are β-hairpin AMPs, while temporin L, pleurocidin, and melittin have an α-helical structure. Despite the differences in their secondary structures, they exhibit a broad spectrum of antimicrobial activity and are also efficient against ESKAPE pathogens and opportunistic pathogenic fungi such as Candida albicans.

Among the peptides under study, protegrin-1 (PG-1) has a low minimum inhibitory concentration (MIC) and exhibits a broad spectrum of activity against various pathogens, including the ESKAPE ones. Therefore, taking into account the high antimicrobial activity of PG-1, we decided to use its amino-acid sequence in the design of a biocontrol agent that is based on methylotrophic yeast P. pastoris.

**Table 1:** Antibiotic activity of a panel of representative highly effective AMPs

Susceptible bacteria	MIC, μg/mL				
	protegrin-1	arenicin-1	temporin L	pleurocidin	melittin
Klebsiella pneumoniae	0.5–4	ND	16	4–8	4–64
Acinetobacter baumanii	0.25	4	4	1–2	0.25–0.5
Pseudomonas aeruginosa	4	2–4	16	16–32	2–8
Staphylococcus aureus	4	2–4	2–4	4–16	1–4
Candida albicans	2	24	8	ND	25

^*^ND – no data; the MIC data were adapted from [21] for protegrin-1; from [22, 23] for arenicin-1; from [24] for temporin L; from [25] for pleurocidin; and from [26-29] for melittin.

**Genetic programming of the yeast**

**Fig. 2:**
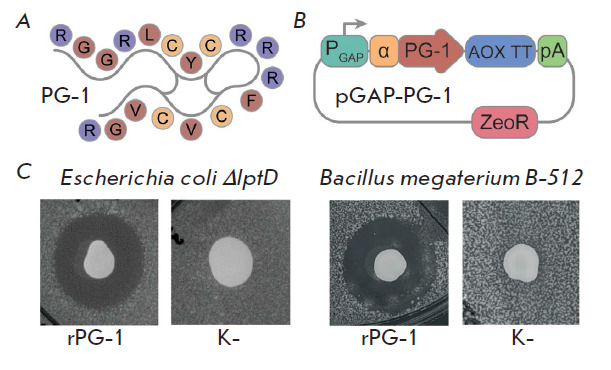
Genetic programming of the *P. pastoris *yeast: (*A*) a structural scheme of protegrin-1, where purple denotes the positively charged amino acid residues, red represents uncharged amino acid residues, yellow represents cysteine residues; (*B*) diagram of the genetic construct for protegrin-1 production in yeast: PGAP – glyceraldehyde-3-phosphate dehydrogenase (GAP) promoter; α – alpha factor signal sequence; PG-1 – codon-optimized protegrin-1 sequence; AOX TT – AOX1 transcription terminator; pA – polyadenylation signal; ZeoR – zeocin resistance; (*C*) test of the antimicrobial activity of the protegrin-producing strain (rPG-1) and control yeast producing the mCherry fluorescent protein (K-). The diameter of the growth inhibition zones was 12 and 14 mm for reporter bacteria *E. coli ΔlptD *and *B. megaterium*, respectively

Protegrin-1 consists of 18 amino acid residues and contains two intramolecular disulfide bonds maintaining the β-hairpin structure (Fig. 2A). Unlike recombinant protegrin, the natural peptide carries an amidated C-terminal arginine residue. The absence of a modification of the C-terminus may affect the stability and activity of AMP; however, efficient in situ production of recombinant protegrin-1 (rPG-1) in a heterologous system can minimize these effects.

The nucleotide sequence of the P. pastoris GS115 protegrin-1 gene was optimized in accordance with the codon frequency and cloned into the shuttle expression vector pGAPZalpha A. The resulting pGAP-PG-1 genetic construct ensured constitutive production of protegrin-1 due to the strong constitutive promoter of the glyceraldehyde-3-phosphate dehydrogenase (GAP) gene, while secretion into the extracellular environment was ensured by the yeast’s alpha factor signal sequence (Fig. 2B). The generated rPG-1 yeast strain, transfected with plasmid pGAP-PG-1, secreted the mature peptide into the extracellular environment, forming distinct zones of growth inhibition of the reporter strains of gram-positive (B. megaterium) and gram-negative (E. coli ΔlptD) bacteria (Fig. 2C).

The level of rPG-1 production by the yeast cells was assessed according to the antimicrobial activity of the culture medium against the reporter bacterium E. coli ΔlptD. A chemical analog of rPG-1 was used as a reference standard. The rPG-1 concentration in the culture medium was 540 ng/mL.

Therefore, we have demonstrated that artificial antimicrobial activity can be reconstructed in rPG-1-producing P. pastoris cells.

**Cocultivation in droplets of microfluidic double emulsion**

Effective biocontrol agents can limit the spread of pathogens they are targeted to. Microbial competition often occurs within certain microcompartments of their habitat, both in soil communities and in the gut microbiome [30]. Hence, when designing biocontrol agents and probiotic organisms, one needs to assess their ability to inhibit the growth of the target bacteria in microcompartments, as well as when bacteria are numerically superior. Droplets of microfluid ic double emulsion make it possible to co-encapsulate effector yeast cells and the reporter bacterial strain in order to evaluate their antimicrobial properties. This model can be further modified to perform large-scale screening of antimicrobial activity.

**Fig. 3:**
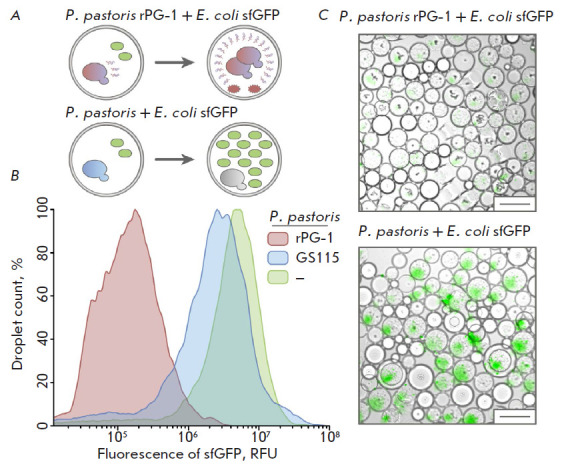
An analysis of the antimicrobial properties of a recombinant biocontrol agent: (*A*) the scheme of cocultivation of effector yeast with a target bacterium in double emulsion drops; (*B*) the results of flow cytometry of droplets after cocultivation: the fluorescence signal distribution is marked with color: for *E. coli *Δ*lptD *sfGFP encapsulated with strain rPG-1, red; with control yeast *P. pastoris *GS115, blue; without yeast, green; (*C*) microfluidic double emulsion droplet microscopy of the target bacterium *E. coli *Δ*lptD *sfGFP encapsulated with effector yeast *P. pastoris *rPG-1c and the control *P. pastoris *GS115. Scale bar: 50 µm

In this study, the recombinant yeast strain producing protegrin (rPG-1) was co-encapsulated with E. coli ΔlptD sfGFP reporter cells constitutively producing the green fluorescent protein sfGFP in microfluidic double emulsion droplets (Fig. 3A). Co-encapsulation of E. coli ΔlptD sfGFP and wild-type yeast (GS115), as well as encapsulation of E. coli ΔlptD sfGFP without yeast, was used as the control. The antimicrobial activity of the recombinant P. pastoris yeast strains was detected according to death or proliferation of the reporter bacterial target and the accompanying sfGFP fluorescence in the microfluidic double emulsion droplets.

After incubation for 24 h, the droplets were analyzed by flow cytometry. Co-encapsulation of the target bacterium and yeast strain rPG-1 reduced the intensity of the fluorescence signal of the reporter compared to that of the droplets containing E. coli ΔlptD sfGFP, either individually encapsulated or co-encapsulated with the control strain GS115 (Fig. 3B). The reduced fluorescence levels in the droplets were indication that growth of the E. coli ΔlptD sfGFP cells in the presence of yeast rPG-1 had been inhibited. Meanwhile, GS115 yeast had no significant effect on the proliferation of E. coli ΔlptD sfGFP, increasing the fluorescence signal in the corresponding droplets.

Microscopic examination of the incubated samples showed highly efficient inhibition of the growth of the E. coli ΔlptD sfGFP reporter strain, accompanied by the proliferation of P. pastoris rPG-1 (Fig. 3C). Meanwhile, droplets filled with the proliferating cells of the reporter bacterium predominated in the case of co-encapsulation of the reporter bacterium and the control GS115 strain (Fig. 3C). A similar effect was achieved when effector yeasts were cocultured with the target bacterium in a 96-well plate. Growth of E. coli ΔlptD sfGFP was inhibited in the P. pastoris rPG-1 suspension, while their growth in the control P. pastoris GS115 suspension was not affected.

Hence, the generated rPG-1-producing yeasts can inhibit the growth of the target bacterium in the coculture within the first day of incubation. These findings can be used for the design of probiotic organisms based on rAMP-producing yeasts and to generate programmable recombinant biocontrol agents.

## DISCUSSION

The rapid spread of antibiotic resistance poses a serious problem in the fight against infectious diseases. The emergence of multidrug-resistant (MDR) bacterial strains further reduces the number of available treatment regimens. Therefore, searching for alternative antimicrobial compounds is an urgent issue. Antimicrobial peptides can become a source of novel antimicrobial agents, since they exhibit activity against a wide range of pathogens, including those associated with multidrug resistance [31].

AMPs include members of various structural classes. Among them, there are β-hairpins, α-helices, as well as linear, combined, and cyclic peptides [32]. The wide structural variability of AMPs allows one to implement different mechanisms of impact on bacterial cells, thus affecting the spectrum of antimicrobial activity. The rational design methods make it possible to fine-tune the physicochemical properties of AMPs and generate a peptide with improved activity and toxicity [33]. Therefore, AMPs constitute a flexible basis for designing effective antimicrobials.

Protegrin-1, which belongs to the β-hairpin AMPs, consists of 18 amino acid residues and contains two intramolecular disulfide bonds. It exhibits broad antimicrobial activity through its interaction with the bacterial membrane and pore formation in it [34, 35]. Taking into account its high antimicrobial activity and the broad range of pathogens susceptible to it, protegrin-1 was selected as an active component for designing a recombinant biocontrol agent.

Heterologous production of AMPs is an important bioengineering issue and also serves as a basis for the design of systems for the large-scale screening of antimicrobial compounds. P. pastoris methylotrophic yeast is widely used in biotechnology, because it allows one to produce recombinant proteins at high yields within short time periods [13, 14]. Generation of recombinant proteins under control of a methanol-inducible alcohol oxidase-1 (AOX1) promoter is the most commonly employed method [36]. However, methanol is easily flammable and a toxic substance; furthermore, induced expression makes it impossible to assess the competitive characteristics of recombinant yeasts in vivo. In our study, the antimicrobial peptide was synthesized in P. pastoris cells under control of a strong constitutive glyceraldehyde-3-phosphate dehydrogenase (GAP) promoter. Therefore, we managed to generate a recombinant yeast strain capable of effectively inhibiting the growth of gram-positive (B. megaterium) and gram-negative (E. coli ΔlptD) bacterial targets.

Biocontrol agents can inhibit the growth of the pathogens they are targeted to [15]. To ensure effective protection against pathogens, biocontrol agents need to be able to compete with these pathogens under conditions of limited resources and space. In this study, such conditions were simulated by microcompartmentalization of the bacterial target and yeast effector in droplets of microfluidic double emulsion and bacterial cells were numerically superior over yeast cells during encapsulation. The yeast strain secreting recombinant protegrin-1 (rPG-1) into the culture medium was found to effectively inhibit the growth of the target bacteria as early as on day 1 after encapsulation. Due to the constitutive production of rPG-1, recombinant yeast exhibits constant antimicrobial activity and can control the growth of microorganisms without the need to add an inducer. Therefore, genetic programming of P. pastoris yeast resulted in the generation of a recombinant biocontrol agent capable of inhibiting the growth of the target bacteria under conditions of competition for space and nutrients.

## CONCLUSIONS

A recombinant biocontrol agent based on methylotrophic yeast P. pastoris, with rAMP protegrin-1 as its active component, has been designed in this study. The resulting yeast strain inhibited reporter target growth both on an agar medium and during cocultivation in droplets of microfluidic double emulsion. The developed strategy for the production of recombinant biocontrol agents is an important stage in elaborating alternative methods for combatting pathogens. Furthermore, the studied approaches can be used to search for novel compounds exhibiting antimicrobial activity by deep functional profiling [37].

## Abbreviations

AMP: antimicrobial peptide
rAMP: recombinant antimicrobial peptide
AR: antibiotic resistance
ESKAPE: a group of pathogens including Enterococcus faecium, Staphylococcus aureus, Klebsiella pneumoniae, Acinetobacter baumannii, Pseudomonas aeruginosa and species of the genus Enterobacter
MIC: minimum inhibitory concentration
PG-1: protegrin-1
rPG-1: recombinant protegrin-1
GAP: glyceraldehyde- 3-phosphate dehydrogenase
sfGFP: superfolder green fluorescent protein
MDR: multidrug resistance
AOX1: alcohol oxidase-1
